# Address before the Optical Society of the State of New York, in Convention at Buffalo, June 17th, 1912

**Published:** 1913-01

**Authors:** Charles F. Prentice

**Affiliations:** New York City; President of the New York State Board of Examiners in Optometry


### Address Before the Optical Society of the State of New York, in Convention at Buffalo, June 17th, 1912

By CHARLES F. PRENTICE, M.E.
New York City
President of the New York State Board of Examiners in Optometry

Mr. Chairman and Fellow Members:

My attendance at this Convention is one of serious duty rather than one of pleasure. Duty, because I have recently expressed opinions in print which surprisingly few of you have indorsed. Evidently there has been misconception of my motive and opinions, but which I believe to be due to subsequent editorial criticism in an unfriendly trade journal.

Therefore I am here to vindicate my position, which, indeed, is a trying one because I am expected to merit the approval of my superiors on the one hand, and of my friends among optometrists on the other.

As a subaltern state officer, it is my first duty to uphold the rules and regulations of the Regents and to respect the judgment of the Directors and Faculty of Columbia University, and for whose established optometry courses I am in a large measure personally responsible.

My own zealous guardianship is, therefore, due to personal interest in some facts which are being generally lost sight of, and which indeed some men in this state are disposed to discredit, possibly through fear that there will not be glory enough for all. Therefore, without any modesty in making liberal use of the personal pronoun, or fear of honorable contradiction, I shall here mention them, lest in my absence it be disputed that I instigated legislation which eventually led to the enactment of the New York State Optometry Law through Mr. Arrington's ability and the support that was given to it by the "Old Guard" and others.

Don't misunderstand me! The honors are all theirs and yours. I am only charged by our medical opponents with the responsibility, and I must, therefore, be the victim for the consequences if they are bad. However, I have worked hard to make them good, and therefore ask your aid.

At the New York Convention in 1908, I suggested the state syllabus, which was then thought to be too high, and yet it was subsequently recommended by the optometry board and approved by the Regents. Later I used my personal influence with the First Assistant Commissioner of the New York State Education Department, who caused the optometry courses to be established in Columbia University.

There are a number of men here who will substantiate these statements, and as you had approved all of these things, it was natural to suppose that you would place your trust in me to advise against anything being done to undermine the established integrity of optometry in this state. Am I right or wrong? (Applause).

Well then! Schemes have recently been advocated to enable optometrists to obtain the degree of Doctor, and which are at best only silly attempts to circumvent established usage and the recognized supervision by the state.

Optometrists who support such schemes are disloyal to the state that has really licensed them to practice a drugless therapy through the use of lenses. They do not realize that their encouragement of a college in another state to issue a doctorship will divert degree-hunting students from attendance at Columbia University, and that the state will thus eventually lose university support of the optometry law.

Moreover, even though a foreign college were not involved, Columbia will probably suppress its optometry courses through medical influence and natural disgust for a class of men who fail to appreciate the true value of academic standards.

Imagine my humiliation upon receiving the following as part of a letter from the Director of the Optometry Courses in Columbia:

"Don't you think now that we are willing to take care of the optometrists, they should advertise in a reasonable way without bringing harm to the cause?"

Gentlemen, this was certainly a subtle reminder of the ingratitude of certain optometrists, and I had to receive it and suffer the reprimand for them, the men who malign me.

Unfortunately some very competent and respected optometrists have unwittingly become converted to the opinion that they are justified in using the title "doctor" through having graduated from some unregistered college. However, such a degree is not recognized in New York, and I am confident that such men will henceforth discontinue the practice.

Every intelligent man knows that the caruncle of a cock does not make him the chanticleer, nor does the possession of a fiddle create the virtuoso. Then why should optometrists make themselves ridiculous by clambering for a degree which is as yet not taken seriously by the Regents of the state? Some people may be deceived by psuedo degrees or counterfeit money, but we should not all strive to adopt such expedients because some men may advocate them.

Lunatics do not know or admit that they are crazy, and optometrists who fail to appreciate the principle upon which academic degrees are acquired in New York, similarly do not realize their own folly in calling themselves doctors. Why did they not first consult the proper authorities at home before promulgating their false conceptions among misinformed men who support them? Because they are carried away with the belief that the title "doctor" is an economic and not a scholastic term; because they, with equally mis-placed confidence, believe that the title "doctor" will bring them dollars instead of doughnuts.

Some of these men have told me with genteel complacency that they would not blame me for deserting them. Why? Because they think I am antiquated, and have served their selfish purpose long enough. They are now simply ready to repudiate their benefactor.

For seventeen years I have suffered the general censure of learned medical men, but let me tell you that I did not silently suffer it for a band of cads, pikers or peddlers; but for a principle, which is now in jeopardy. It is regrettable indeed that there are optometrists who lack the required moral rectitude to live up to Governor Hughes' admonition to "preserve a proper *esprit de corps*." Little did the Governor then suspect that he might be casting pearls before swine.

Rest assured, gentlemen, that it takes something more than self-interest to bear the abuse of one's intelligence and moral character by learned medical men while remaining loyal to what they have considerately called "bad company."

Of course, there are many conscientious and able men in this society who have my greatest respect, and with some of whom I have for many years had a most intimate and friendly acquaintance. It is to them I now appeal for support, in order to prove to interested communities that we are not all of the deadly stripe painted before the Regents, the Directors of Columbia University and the medical profession at large.

It is for the people and the better class of optometrists that I preached the doctrine that the practice of optometry shall be founded upon optical knowledge, but not with the assumption that they should never be required to qualify at least to some degree in medicine.

Now, as to my recently published utterances respecting the education of future eye practitioners, I will repeat my opinion that the public will receive the best, the most comprehensive eye-service from those practitioners who have at least as much knowledge of medicine as they are now required among us to have in optics. Even if only this should result, their medical knowledge will be as far from being complete as their required knowledge of optics, which according to the state syllabus, is specifically limited. This is why I have for years compared optometry with dentistry, on belief that "all future practitioners of optometry should be graduate physicians."

However, it is not your business, nor is it my business, to say how such medical knowledge should be injected into optometry. There is not an optometrist in the land who is sufficiently versed in medicine, either by collegiate education or amateur experience, to prescribe the required scope of medical education that should be given to the more advanced practitioner of optometry. The optometrist having such presumption would be rudely encroaching upon the medical perrogative.

I have simply asserted my belief that medical education should be included in the optometry courses of future eye practitioners, while expecting fair-minded and competent medical authorities to determine its limitations. Moreover, even should this eventuate, established optometrists will not suffer through such requirement any more than those men who have failed to qualify in theoretic optics as now required by law.

Gentlemen, you must be prapared to raise and not to lower the standard of education. Remember, I am only asking you to be prepared to show a disposition of fairness to arbitrate among collegiate men, to concede that your standard may be improved upon, so that when the time comes you will be prepared to give and take in the effort of commendable uplift. You must put yourselves on record as being opposed to the tactics of "bad company."

Put yourselves in a position to honorably arbitrate when your optometry courses in Columbia are threatened with suppression. You should be progressive, not retrogressive. Nor will standpatism suffice, because you must not abide in the faith that Columbia University is going to stand pat for you very long. The opinions of learned medical men will have far greater influence with the directors of Columbia University than those of self-styled "optometric doctors" who are already filling them with disgust. Columbia is not going to stand sponsor for their irritating and wanton behavior.

The opinions of men in Pennsylvania, Ohio and other states are not altogether applicable in New York, where we are under supervision of the Regents, who look at things from an academic point of view and who are not interested in the regulation of selfish commercialism, but of ethical professions. Show also the Regents that you are worthy of their respect.

Where among you are the men who do not desire to support the Regents, the Directors and the Faculty of Columbia University? And what better policy than is here outlined can you advocate to prove your loyalty to these institutions in the state? I know you will need their friendly support even to a greater degree in the very near future.

Is this not sufficient warning from one who, in the very nature of things, must be better informed on the inner situation than the critic who is most popular for his use of a slanderous tongue? Support my policy, and you will be altruistically helping the cause of optometry, and incidentally yourselves through gaining the respect of learned men. No greater harm can come to you than to myself by such action. You will certainly, from a proper appreciation of my past services, lend a deaf ear to the critics who have set themselves up as the supreme judges of criminality in their own court. Recently advocated retrogressive schemes have compelled me to reveal my own unbiased opinions, and simply because I can also no longer stand sponsor for men who are recklessly endangering the established integrity of the optometry law of New York. These men have precipitated a crisis so far-reaching in its detrimental results that I am confident that you will approve of my loyal performance of duty in defending the state's interest in optometry.

Our past accomplishments should commend themselves to every intelligent observer. We have closed the door against a continuance of postulate optical practice among optometrists. As a result, we have only had thirty-five men, qualified by examination, enter the field of practice during the past four years; whereas, if we had not had an optometry law we should undoubtedly have had three hundred or more additional incompetents working among us today.

Eighteen years ago I suggested legislation which, through the efforts of others in twenty-seven states, upholds the constitutional right of optometrists. At that time I had no other object in view.

Eighteen days ago I made another suggestion—the one repeated to you today—which, if unheeded, may cause us to lose much that we have gained in the State of New York.

We have simply reached another crisis wherein my judgment is again criticised; yet you, as well as the Regents and Columbia University, have in the past approved it, and a few of you have profited exceptionally by it; notably examined men in New York, some of whom are making my position very difficult.

Therefore, it ought to be fair risk to again follow my advice, now that you have heard the reason for it.

Note by the Editor: It is scarcely necessary to say that the publication of a communicated article does not imply editorial agreement. In fact, as a general policy, we give the preference to articles which present minority views, lead to critical investigation and thus, whether themselves right or wrong, ultimately tend to establish the truth. For the same general reason, we feel that it is proper to publish articles, occasionally, by men not themselves members of the medical profession. The open-minded, judicial attitude of according at least a respectful hearing to both sides of any controversy, whatever our preconceived views may be, is really the highest ethics. We are authorized to state further that the acceptance of the present article has been recommended by others, including the author of the next paper. We therefore ask the careful reading of both papers, without prejudice, and such further discussion of the problems presented, as may occur to our readers.

